# A commentary on ‘ChatGPT in medicine: prospects and challenges: a review article’ – correspondence

**DOI:** 10.1097/JS9.0000000000001487

**Published:** 2024-04-19

**Authors:** ArunSundar MohanaSundaram, Talha Bin Emran

**Affiliations:** a School of Pharmacy, Sathyabama Institute of Science and Technology, Chennai, Tamilnadu, India; b Department of Pharmacy, Faculty of Allied Health Sciences, Daffodil International University, Dhaka, Bangladesh

*Dear Editor,*

In this Digital Epoch of Medicine, a plethora of papers exploring diverse applications and challenges of ChatGPT (Generative Pre-trained Transformer), a generative artificial intelligence (AI) chatbot from OpenAI, have been published. In this milieu, Tan *et al*.^1^ published an article in this journal about the prospects and constraints of espousing ChatGPT in medicine.

The authors have comprehensively reviewed the potential use of ChatGPT for diverse medical applications, including the transformation of consultation support for patients, simplifying clinical assessment and workflow management, providing medical education and training, and facilitating clinical research. Besides, constraints in deploying ChatGPT that are pertinent to legal, ethical, and technical aspects were also discussed^1^. However, various potentially commendable and challenging facets of ChatGPT’s deployment in the medical field have been overlooked. In this communication, we have discussed the utility of ChatGPT in assessing drug interaction, drug discovery and development, evaluating scientific transparency, mitigating infodemic, handling multimodal data, and non-inclination to racial and ethnic disparity. Furthermore, concerns regarding over-reliance on ChatGPT and its inappropriate responses to general public inquiries were also discussed.

First, drug interaction, one of the most essential aspects of medicine hasn’t been discussed in the review by Tan *et al*.^1^. Recently, Al-Ashwal *et al*.^2^ showed that ChatGPT and other AI chatbots were able to investigate potential drug adverse effects and interactions with good sensitivity. However, ChatGPT-4 displayed inferior performance (when compared to other AI chatbots like Bing AI and Google’s Bard) in terms of accuracy and specificity in drug interaction analysis. In another study, ChatGPT demonstrated good predictive and explanatory capacity for common drug–drug interactions (DDIs). Nevertheless, around half of the outputs about DDIs were ‘inconclusive’^3^. Considering such a lower degree of reliance on ChatGPT-based DDI information, cautious use of such information is obligatory. In a study assessing the potential for drug–herb interactions, ChatGPT displayed a higher appropriateness rate (61%) for public drug consultation questions than those posed by healthcare professionals (39%)^4^.

Second, harnessing the potential of ChatGPT in drug discovery and development is another overlooked yet a key area in medical research. Recently, Chakraborty *et al*.^5^ have discussed the potential use of ChatGPT in accelerating drug discovery and development. Drug discovery is an arduous journey – ranging from targeted drug designing and lead identification to formulation and clinical development – spanning at least a decade and a whopping $2.6 billion investment to launch a single drug in the market^6^. In this decade, there has been a more than 27-fold increase in investment (counting to $60.2 billion) by 800 AI-driven pharma and biotech companies as of March 2023. Potential applications of AI-driven technologies in drug discovery include early drug development, drug repurposing, end-to-end drug development, pre-clinical and clinical development, etc. The day when AI-enabled ChatGPT finds its role in an array of next-generation drug discovery processes is not so far. There are noteworthy breakthroughs in AI-driven technologies including Deep Genomics, DeepMind’s AlphaFold, In silico Medicine’s GENTRL, Peptilogics, etc., that predict molecular targets, design novel drug candidates, and predict protein’s 3D shape using amino acid sequence. Recently, Exscientia, a British pharmatech startup and the first full-stack AI drug discovery company, has announced that its ‘sixth’ AI-designed molecule is set to enter clinical trials^7^. Wang *et al*.^8^ demonstrated the design of various multi-target anti-cocaine addiction leads by using ChatGPT.

Third, assessment of drug interactions is another lucrative application of ChatGPT. With a global prevalence of 37.2%, multimorbidity (multiple diseases/disorders) among the adult population is a growing concern worldwide^9^. Considering the increasing prevalence of polypharmacy (multiple medications), the need to better understand the drug adverse effects, drug–drug interactions, drug–herb interactions, drug–diseases interactions is critical for not only physicians but also other healthcare providers (pharmacists, nurses, nutritionists, etc.), patients, and caregivers. With its enormous potential to handle a high volume of electronic health records (EHR) and genetic data, ChatGPT can predict/assess diverse adverse effects of drugs including various drug interactions.

Fourth, lack of transparency and bias in scientific publications cast doubt on the integrity of medical research. Assessment of conflicts of interest (COI) data using the ‘Open Payments’ database is being used to maintain transparency in the financial ties between scientific researchers and the pharmaceutical, biotechnology and medtech industries. In this line, ChatGPT was found to be a reliable automated tool to analyze potential COI data in published papers^10^.

Fifth, ChatGPT has the potential to provide factual health information, thereby limiting the spread of fake information. For example, infodemic – a neologism of ‘information’ and ‘epidemic’ – is a serious public health threat and a key reason underpinning vaccine hesitancy. ChatGPT could be effectively used as a potential tool to combat vaccine hesitancy and enhance the trust in vaccines^11^.

Sixth, the evolution of the futuristic multimodal chatbot (ChatGPT-4) makes it possible to handle images, videos, audio, and complex textual data. Until the launch of ChatGPT-4 in March 2023, ChatGPT handled only unimodal (text-based mode) data. Hence, ChatGPT acts as a promising avenue that bridges healthcare providers and patients^12^.

Seventh, potential racial and ethnic disparity (RED) in handling healthcare data has been a lurking concern. To assess the risk of such bias in deploying ChatGPT for the analysis of electronic health records (EHR), Hanna *et al*.^13^ have analyzed the EHR data of 100 deidentified HIV patients and found that ChatGPT didn’t show any significant racial/ethnic bias.

While ChatGPT ushered in an era of revolutionary mass utilization of generative AI in diverse fields, some people deem that the genie of ChatGPT and other LLMs (large language models) is out of the bottle, and we couldn’t stall the repercussions. Over-reliance on ChatGPT could be a perilous trend in the healthcare arena. According to an expert survey conducted in 2022, Human-Level Machine Intelligence (a.k.a. Artificial General Intelligence) would not be available until 2059^14^. In a study conducted by Japanese researchers on inquiries about over-the-counter (OTC) drugs by the general public, the responses of ChatGPT were found to be less accurate and less reliable^15^. Furthermore, only 21% of the responses met all the three criteria involving coherence, correctness, and appropriateness. The reproducibility of responses was only 62% when the same inputs were assessed on another day. Beyond a shadow of a doubt, ChatGPT could be used as a next-generation virtual assistant in medicine with umpteen benefits, but the responses provided should be validated not only in terms of coherence but also in terms of accuracy and completeness.

## Ethical approval

Not applicable.

## Sources of funding

No funding was received.

## Author contribution

A.S.M.: conceptualization, writing, revision, and supervision; T.B.E.: data collection, revision, and supervision.

## Conflicts of interest disclosure

The authors declare that they have no conflicts of interest.

## Research registration unique identifying number (UIN)

1. Name of the registry: not applicable.
2. Unique identifying number or registration ID: not applicable.
3. Hyperlink to your specific registration (must be publicly accessible and will be checked): not applicable.

## Guarantor

Talha Bin Emran.

## Provenance and peer review

Not commissioned, internally peer-reviewed.

## Data availability statement

The data in this correspondence article is not sensitive in nature and is accessible in the public domain. The data is, therefore, available and not of a confidential nature.
